# Subcellular Nutrient Element Localization and Enrichment in Ecto- and Arbuscular Mycorrhizas of Field-Grown Beech and Ash Trees Indicate Functional Differences

**DOI:** 10.1371/journal.pone.0114672

**Published:** 2014-12-08

**Authors:** Jasmin Seven, Andrea Polle

**Affiliations:** Forstbotanik und Baumphysiologie, Büsgen-Institut, Büsgenweg 2, 37077 Göttingen, Germany; Friedrich Schiller University, Germany

## Abstract

Mycorrhizas are the chief organ for plant mineral nutrient acquisition. In temperate, mixed forests, ash roots (*Fraxinus excelsior*) are colonized by arbuscular mycorrhizal fungi (AM) and beech roots (*Fagus sylvatica*) by ectomycorrhizal fungi (EcM). Knowledge on the functions of different mycorrhizal species that coexist in the same environment is scarce. The concentrations of nutrient elements in plant and fungal cells can inform on nutrient accessibility and interspecific differences of mycorrhizal life forms. Here, we hypothesized that mycorrhizal fungal species exhibit interspecific differences in mineral nutrient concentrations and that the differences correlate with the mineral nutrient concentrations of their associated root cells. Abundant mycorrhizal fungal species of mature beech and ash trees in a long-term undisturbed forest ecosystem were the EcM *Lactarius subdulcis*, *Clavulina cristata* and *Cenococcum geophilum* and the AM *Glomus* sp. Mineral nutrient subcellular localization and quantities of the mycorrhizas were analysed after non-aqueous sample preparation by electron dispersive X-ray transmission electron microscopy. *Cenococcum geophilum* contained the highest sulphur, *Clavulina cristata* the highest calcium levels, and *Glomus*, in which cations and P were generally high, exhibited the highest potassium levels. *Lactarius subdulcis*-associated root cells contained the highest phosphorus levels. The root cell concentrations of K, Mg and P were unrelated to those of the associated fungal structures, whereas S and Ca showed significant correlations between fungal and plant concentrations of those elements. Our results support profound interspecific differences for mineral nutrient acquisition among mycorrhizas formed by different fungal taxa. The lack of correlation between some plant and fungal nutrient element concentrations may reflect different retention of mineral nutrients in the fungal part of the symbiosis. High mineral concentrations, especially of potassium, in *Glomus* sp. suggest that the well-known influence of tree species on chemical soil properties may be related to their mycorrhizal associates.

## Introduction

Trees can be considered as ecosystem engineers because they influence soil properties and belowground community structure by species-specific effects, which in turn may have feedback effects on tree functions and vegetation composition [1]–[6]. In temperate forests, important dominant stand-forming species with different life history traits of nutrient, water and light use efficiency as well as of different litter quality, root morphology and mycorrhizal associations are beech (*Fagus sylvatica* L.) and ash (*Fraxinus excelsior* L.) trees [7]–[9]. For instance, beech litter has a high C-to-N ratio >50, a high lignin content and a slow decomposition rate, whereas ash produces high quality litter with a low C-to-N ratio <30, a low lignin content and a fast decomposition rate [10], [11]. In roots of these tree species nutrient element concentrations differ, with higher concentrations of phosphorus (P), sulphur (S), potassium (K) and magnesium (Mg) and lower concentrations of calcium (Ca) in ash than in beech [12]. Beech roots are colonized by ectomycorrhizal (EcM) fungi and those of ash by arbuscular mycorrhizal (AM) fungi [13]. These symbiotic fungi differ in nutrient acquisition strategies; EcM appear more efficient for host nitrogen and AM for P supply [14]. Although mycorrhizal fungi have profound effects on plant nutrient acquisition, very little information is available on the role of AM for tree nutrition in the temperate region.

The nutrient element concentrations in roots and their distribution between fungal and plant structures can inform on interspecific differences of mycorrhizal access to soil nutrient elements. Under controlled conditions, electron microscopy combined with energy-dispersive X-ray-microanalysis (EDX) has often been applied to determine the distribution of nutrient elements between root and fungal cells of EcM (e.g., [15]–[22]). For example, under P starvation, but not with sufficient P supply EcM with *Suillus bovinus* increased P in *Pinus sylvestris* roots cells compared to non-mycorrhizal plants [16]. In contrast to the plant, the intracellular P concentrations of the fungus were unaffected by external P concentrations [16]. Potassium, which is mainly localized to cell walls and cytosol of root cells, was not affected by fungal colonization [17]. P containing storage granules were found together with Mg and K in fungal vacuoles using EDX-TEM (transmission electron microscopy) [15], [19], [23], [24].

Only in few studies, element concentrations were determined in forest samples of EcM (e.g., [25]–[28]. These studies showed high individual variation of nutrient elements in the outer fungal mantle of *Lactarius*-beech EcM samples ([26] by EDX scanning electron microscopy), interspecific differences in the element concentrations in the outer mantle of a range of typical beech and pine-colonizing fungi ([27], [28] by inductively coupled plasma-atomic emission spectrometry (ICP-AES) and electron energy-loss spectroscopy (EELS)) as well as in whole EcMs encompassing root and fungal tissues ([25] by ICP-AES). Significant correlations between S, Mg or Ca concentrations in EcM (by ICP-AES) and in the fungal mantle (by EELS) were detected, but not for P and K [28]. It was, therefore, assumed that the distribution of the latter nutrient elements between the outer cell layer of the hyphal mantle and the whole EcM is uneven [28]. This suggestion has not yet been further investigated. To increase our knowledge on the functions of different mycorrhizal species that coexist in the same environment, it is important to know whether the concentrations of nutrient elements in fungal cells exhibit interspecific differences and whether these potential differences were reflected in different nutrient levels in the root cells.

In the present study we investigated the distribution of nutrient elements between fungal and plant structures in EcM and AM-colonized root tips of mature beech and ash trees that grow in a long-term undisturbed, deciduous forest (National Park Hainich, Germany). Mycorrhizal fungal species richness has been characterized previously, showing that common EcM species of beech such as *Lactarius subdulcis*, *Cenococcum geophilum* and *Clavulina cristata* [25], [27], [29], [30] are also abundant in the Hainich forest [13]. *Glomus* spec. were found to be the main AM fungi on ash trees in this ecosystem [13]. Here, we analysed the localization of P, S, Mg, Ca, and K in root cells (vacuole, cell wall) in EcM of beech with *Clavulina cristata, Lactarius subdulcis* and *Cenococcum geophilum* (walls and cell lumina of the hyphal mantle and the Hartig net) and in *Glomus* spec. (arbuscules, intra- and intercellular hyphae) on ash. We hypothesized that (i) the EcM species on beech differ in mineral nutrient concentrations, (ii) AM fungi accumulate higher nutrient element concentrations than EcM and (iii) that these differences are reflected in different mineral nutrient concentrations in the root tissues.

## Materials and Methods

### Ethics statement

Permission for sampling in the state-owned National Park Hainich (Thurigina, Germany) was granted by the National Park Administration (Thuringia, Germany according to § 72 BbgNatSchG). No protected species were sampled.

### Sampling and fungal material

Samples were collected in the National Park Hainich (Thuringia, Germany) at the 6^th^ of November 2008 in a mixed forest near the Thiemsburg (51°06′N, 10° 31′E), with a basal area of *Fagus sylvatica* of 16.6 m^2^ ha^−1^ and of *Fraxinus excelsior* of 12.8 m^2^ ha^−1^ [31], [32]. The soil at this site is characterized as Luvisol with parent material of Triassic Limestone covered with Loess. The soil pH (H_2_O) was 5.3, and the main nutrient concentrations in soil were 27.8 mg C g^−1^ dry mass, 2.0 mg N g^−1^ dry mass and 0.4 mg P g^−1^ dry mass. The mean annual temperature is 7.5°C and the mean annual precipitation is 670 mm. Detailed information about the study area can be found in [33], [34]. Nine soil cores containing roots of ash and beech were sampled with a soil corer (8×20 cm), transported immediately into the laboratory and processed the same day. Root tips were cleaned from adhering soil by tooth picks and by brief washing in water. Roots of beech and ash were distinguished under a dissecting microscope (Stemi SV 11, Zeiss, Jena, Germany).Beech root tips colonized with *Cenococcum geophilum*, *Lactarius subdulcis* or *Clavulina cristata* were collected as described previously [13].; online resource goe-fungi http://www.uni-goettingen.de/de/goe-fungi/92389.html; accession numbers EU346870; EU346875; EU816621). Because an individual beech root in a soil core hosted several, but not necessarily all fungi that were included in this study, roots from nine cores were inspected and one to two roots per core were required to obtain n = 6 biological replicates of each ectomycorrhizal fungal species. A biological replicate was an ectomycorrhiza with a given fungal species on an individual root. From ash roots, approximately 30 root tips were sampled from roots in different soil cores.

### Preparation of root tips for TEM-EDX microanalysis

Fresh root tips of beech and ash were transferred into a small mesh and rapidly frozen in a mixture of propane:isopentane (2∶1) cooled in liquid nitrogen. The samples were freeze-dried at −45°C for 3 days and stored dry over silica gel until further processing. Freeze-dried root tips were vacuum-pressure infiltrated with diethyl-ether (Merck, Darmstadt, Germany), and embedded with stepwise increasing concentrations in styrene-methacrylate (Merck, Darmstadt, Germany; [35]). The samples were embedded in gelatin capsules in 100% plastic. All steps of tissue processing were carried out under water-free conditions to prevent displacement or loss of diffusible elements in the root tissue. Polymerization of the plastic-filled capsules took place in an oven at 60°C overnight and at 45°C for 10 days. After polymerization, semi-thin (1 µm) sections of the root tips embedded in plastic capsules were cut using an ultramicrotome (Ultracut E, Reichert-Jung, Vienna, Austria) with dry glass knives. The sections were mounted with an adhesive as described by [36] on hexagonal copper grids (Athene, provided by Plano, Wetzlar, Germany), coated with carbon and stored over silica gel.

### Staining with Toluidine Blue and light microcscopy

For the detection of mycorrhizal structures, semi-thin sections of root tips were cut from the same blocks used for X-ray microanalysis, stained with Toluidine Blue (Merck, Darmstadt, Germany), mounted with Euparal (Carl Roth KG, Karlsruhe, Germany) on glass slides and visualized under the light microscope (Zeiss, Axioplan, Oberkochen, Germany). Sections were photographed with a digital camera (Zeiss, AxioCam MrC, Software AxioVision Release 4.6.3).

### Scanning transmission electron microscopy (STEM) and X-ray microanalysis (EDX)

Semi-thin sections were analyzed by EDX microanalysis (EDAX DX-4, EDAX International Mahwah, NJ) under standardized conditions with a FEI Tecnai G^2^ Spirit BioTWIN transmission electron microscope (TEM; FEI Company, Eindhoven, The Netherlands) as described by [37]. The microscope was equipped with a Si (Li) detector with a thin beryllium window (8 mm thick).

Point measurements as well as spectral images (mappings) using the scanning transmission electron microscopy mode (STEM) were conducted in a matrix of 40×40 measurement points. The dwell time for each measurement point was 10.000 ms in the live second mode. The take-off angle was 15° tilt towards the detector. The accelerating voltage was 80 kV.

The spectra were analyzed using the Tecnai Image Analysis (TIA)-Offline software (FEI Company, Eindhoven, The Netherlands). The spectra were extracted at selected positions in cross sections and automatically background fitted by the TIA-Offline software. Relative element concentrations are indicated as background-fitted peak intensities. EDX spectra were collected between 0 and 10 keV. The peak centers of the different elements (K alpha) are Mg 1.25 keV, P 2.01 keV, S 2.31 keV, K 3.31 keV, and Ca 3.69 keV). Calibrations of the method show that net peak heights are linearly correlated with the concentrations of the target element in agar standards [35]–[37]. N was not included because the N peak is masked by the huge carbon peak.

The relative element abundances in cross sections of plant roots and fungi were analyzed in cell walls and vacuoles of EcM fungal cells in the hyphal mantle and in the Hartig net, for AM fungi in intercellular hyphae, intracellular hyphae and arbuscules as well as in the plant root cell walls and vacuoles adjacent to the fungal cells. Ten replicates were investigated in each of these compartments in six different biological root samples of each ash and beech (n = 60). All statistical analyses were conducted with the program Statgraphics Centurion (St Louis, Mo). When necessary, data were logarithmically transformed to satisfy the criteria of normal distribution and homogeneity of variance. Data are means (± SE). Analysis of variance and a post hoc test (LSD) were used to detect significant differences at p≤0.05. Principle component analysis (PCA) and Generalized Linear models (GLM) were calculated with Statgraphic Centurion (St Louis, Mo).

## Results

### Mapping of the subcellular element distribution in EcM and AM

**Figure 1:**
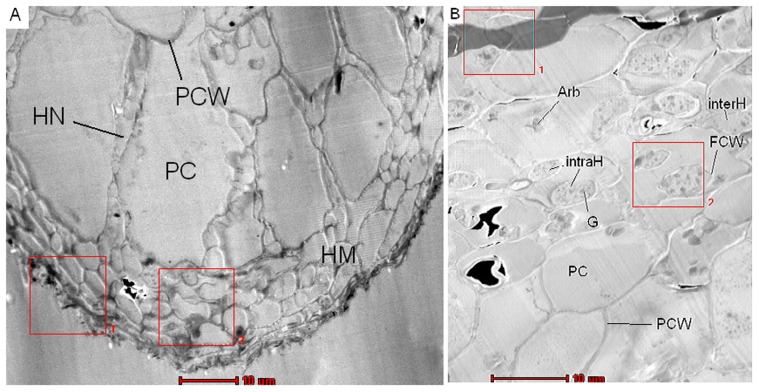
Cross section of beech (A, *Fagus sylvatica*) and ash (B, *Fraxinus excelsior*) mycorrhizal root tips. The beech root was colonized by *Lactarius subdulcis* and the ash root by *Glomus* sp. PCW  =  plant cell wall, HN  =  Hartig Net, PC  =  plant cell, FCW  =  fungal cell wall, HM  =  hyphal mantle, Arb  =  arbuscles, intraH =  intracellular hyphae, interH  =  intercellular hyphae. Bar = 10 µm. Area 2 (red square)  =  area for EDX-STEM mapping with 40×40 measurement points. Area 1 was used for drift correction.

**Figure 2:**
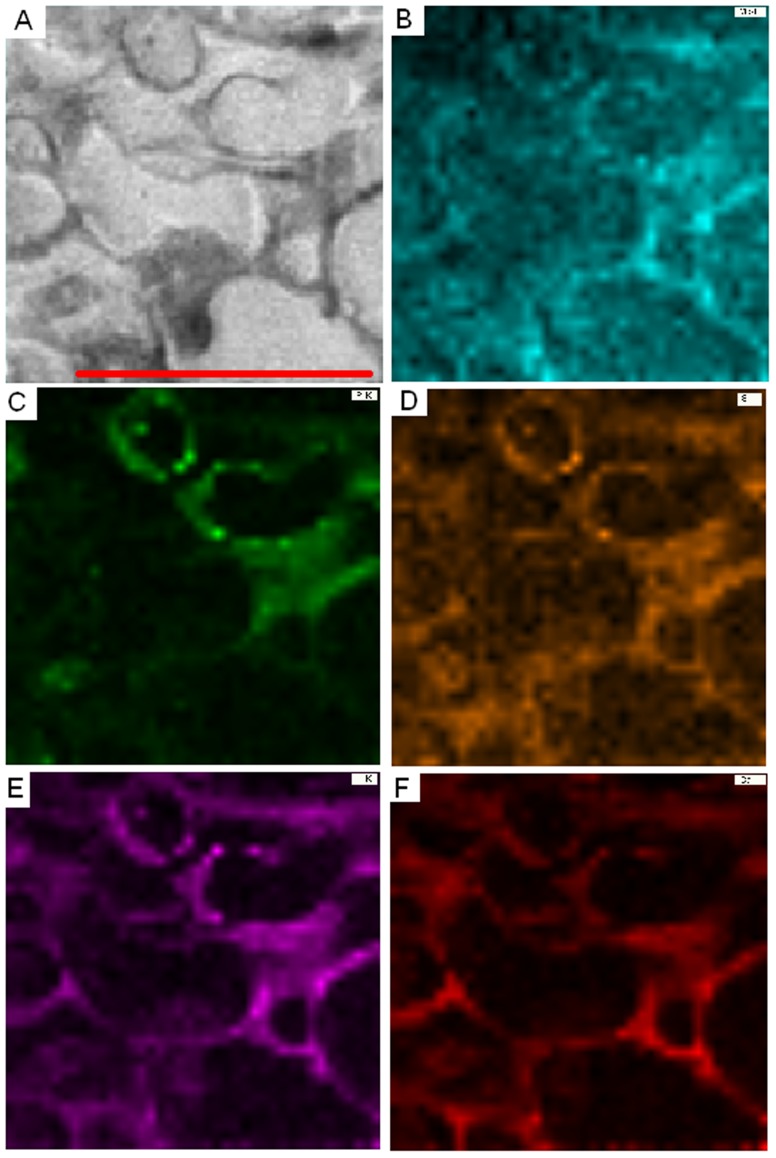
EDX-STEM-mapping of an ectomycorrhizal root of beech (*Fagus sylvatica*) colonized by *Lactarius subdulcis*. EDX-STEM-mapping of 40×40 measurement points in the area depicted (A) of magnesium (B, turquoise), phosphorus (C, green), sulphur (D, orange), potassium (E, purple), and calcium (F, red). Scale bar: 10 µm.

For the analysis of the subcellular element distribution typical cross sections of EcM and AM structures as depicted in Fig. 1 were chosen. EcM on beech roots are characterized by a hyphal mantle, in which interwoven fungal hyphal cells ensheath the root tip (HM; Fig. 1A). Between the root cells, fungal cells forming the Hartig net can be detected (HN, Fig. 1A). An example for EDX-STEM mapping in the hyphal mantle of *Lactarius subdulcis* adjacent to root cells (see frame in Fig. 1A) is shown in false colors for P, S, K, Mg and Ca (Fig. 2). Mg was relatively evenly distributed across the fungal cells and showed only slight enrichment in the fungal cell walls (Fig. 2B). P was more enriched in cells towards the inner side of the mantle and some intensely stained P-containing granules were observed in the cells (Fig. 2C). In some of these granules, which could also be detected by their dark staining in the STEM image (Fig. 2A), S and K were also present (Fig. 2D,E), whereas Ca was not detected in them (Fig. 2F). In general, the measured elements were more enriched in the cell walls than inside the cell and this effect was stronger for K and Ca than for Mg and S (Fig. 2).

**Figure 3:**
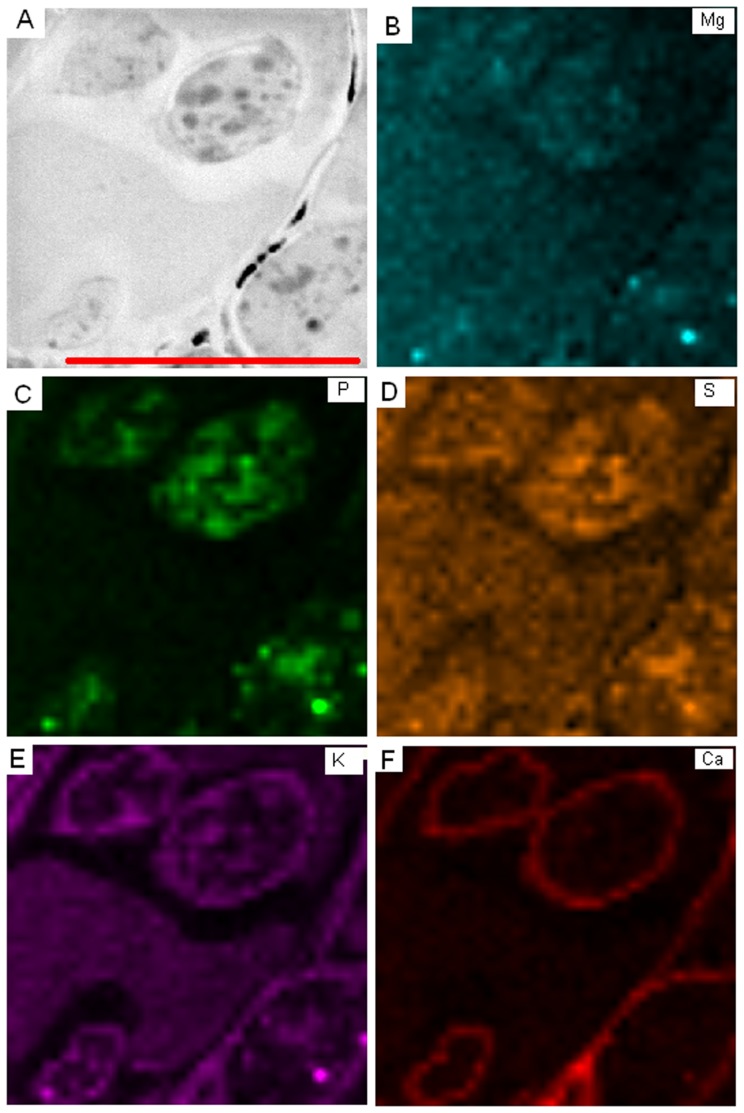
EDX-STEM-mapping of arbuscular mycorrhizal root of ash (*Fraxinus excelsior*) colonized by *Glomus* sp. EDX-STEM-mapping of 40×40 measurement points in the area depicted (A) of magnesium (B, turquoise), phosphorus (C, green), sulphur (D, orange), potassium (E, purple), and calcium (F, red). Scale bar: 10 µm.

AM of ash roots were characterized by arbuscules as well as by inter- and intracellular hyphae localized inside the roots (Fig. 1B). A representative EDX-STEM mapping showed that Mg was relatively homogeneously distributed across root cells and fungal structures (Fig. 3A,B). The intracellular hyphae and arbuscules were strongly enriched in P (Fig. 3C). Strong P accumulation, evident from the intensely green coloured granules in the fungal cells, co-localized with Mg, S and K (Fig. 3B,D,E), but not with Ca (Fig. 3F). In contrast to P and Ca, relatively high levels of S and K were also detected in ash root cells (Fig. 3D,E). It was notable that K and Ca were clearly enriched in the cell walls of plant origin as well as in cell walls or in the periplasmatic space surrounding the intracellular fungal structures (Fig. 3E,F). Ca levels inside root and fungal cells of AM were low (Fig. 3F).

### Spatial and taxon-related variation in the relative nutrient element concentrations in AM and EcM

**Figure 4:**
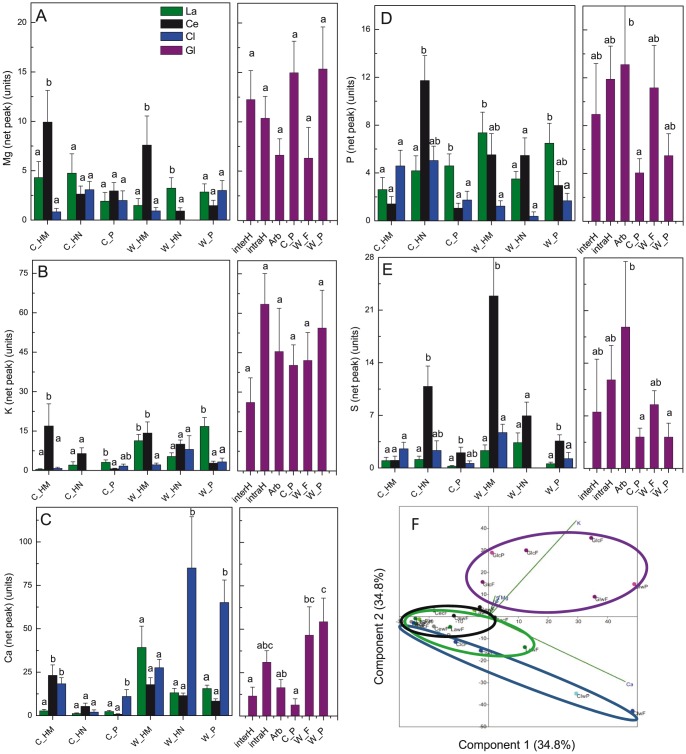
Subcellular nutrient element levels in different subcellular structures of mycorrhizal roots associated with different mycorrhizal taxa [(A) Magnesium, (B) Potassium, (C) Calcium, (D) Phosphorus, (E) Sulphur] and principle component analysis (F). Data indicate means (n≥20 to 60±SE). Different letters for a given structure (EcM) or between the different compartments (AM) indicate significant differences at P≤0.05. La  =  Lactarius subdulcis, Ce  =  Cenococcum geophilum, Cl  =  Clavulina cristata, Gl  =  Glomus sp., C  =  cell, W  =  Wall, HM  =  Hyphal mantle, HN  =  Hartig net, InterH  =  intercellular hyphae, intraH  =  intracellular hyphae, Arb  =  arbuscules, F  =  fungus, P  =  plant.

To obtain semi-quantitative information on nutrient element concentrations in different subcellular locations of different symbiotic fungi and the colonized root tips, peak heights were extracted from vacuoles and cell walls of root and fungal cells of root tips colonized with *Cenococcum geophilum*, *Lactarius subdulcis*, *Clavulina cristata* or *Glomus* sp. (Fig. 4A-E). The analysis of the baseline-corrected peaks revealed significant differences for nutrient elements in a given subcellular compartment among the studied EcM fungi and the root cells associated with that fungal taxon, but also strong variation of the element levels in different compartments (Fig. 4A-E). The AM generally contained high Mg and K levels (Fig. 4A,B) and hyphae and arbuscules were enriched in P and S and relatively depleted in Ca compared with cell walls of the fungal or root cells (Fig. 4D,E).

To find out whether different mycorrhizas can be distinguished based on the relative nutrient element levels at the subcellular scale, we conducted PCA (Fig. 4F). The PCA explained >90% of the variation by two components. PC1 was mainly determined by Ca and K (positive loadings of 0.84 and 0.54) and PC2 by positive loadings for K (0.79) and negative for Ca (-0.54). PC2 separated the data for ash/*Glomus* from those of beech/EcM colonized roots with positive K loadings as the main factor. The profile most divergent from *Glomus* was that of *Clavulina* located in the negative range of PC1 and PC2 with Ca as main factor. The Ca effect was strongest in cell walls of both plant and fungal cells of *Clavulina*. *Cenococcum* showed the least variation among the different structures analyzed and the data were positioned along PC1 in the negative range, where no distinct factor was identified. *Lactarius* assumed an intermediate position between *Clavulina* and *Cenococcum*, probably because it accumulated also relatively high Ca levels in fungal walls. The PCA did not reveal separation of plant and fungal structures. Plant structures (cell walls and cells) were positioned in the range of the data of their respective fungal symbionts.

To investigate whether the interspecific differences identified by PCA for the fungi were significant at the level of distinct nutrient elements, we compared means of across all fungal structures (Table 1). *Glomus* exhibited higher Mg, P, and K concentrations than any other of the analyzed EcM species. Among the tested EcM species, *Cenococcum* contained the highest concentrations of Mg, P, and K, and *Clavulina* the lowest (Table 1). In *Cenococcum* and *Glomus* high S levels were present (Table 1). In *Clavulina* and *Glomus* high Ca levels were detected (Table 1).

**Table 1:** Relative abundance of nutrient elements in plants and their associated fungal species.

Tissue	Symbiont	Mg	P	S	K	Ca
Fungus	Lactarius	3.40±0.70b	4.44±0.61a	2.02±0.44a	4.94±0.86a	14.42±3.41a
Fungus	Cenococcum	3.81±0.75b	7.09±0.99b	9.70±1.48b	10.36±1.70b	11.93±1.43a
Fungus	Clavulina	1.04±0.22a	2.84±0.57a	3.04±0.57a	2.12±0.73a	28.07±4.86b
Fungus	Glomus	8.68±1.27c	11.35±2.06c	11.97±3.22b	43.62±6.49c	26.07±5.02b
Plant	Lactarius	2.38±0.61a	5.54±0.97b	0.41±0.12a	9.93±1.84b	8.95±1.16a
Plant	Cenococcum	2.21±0.50a	2.00±0.62a	2.81±0.55b	1.70±0.41a	4.52±0.79a
Plant	Clavulina	2.49±0.70a	1.70±0.48a	0.93±0.44a	2.46±0.82ab	38.07±7.57b
Plant	Glomus	15.15±2.64b	4.77±1.08ab	4.19±1.08b	47.32±8.13c	30.35±7.64b
P_(fungus/plant)_	Lactarius	**0.028**	0.162	**<0.001**	0.096	0.46
P_(fungus/plant)_	Cenococcum	0.525	**<0.001**	**<0.001**	**<0.001**	**<0.001**
P_(fungus/plant)_	Clavulina	0.198	**0.011**	**0.002**	0.895	0.534
P_(fungus/plant)_	Glomus	0.344	**0.001**	0.078	0.639	0.097

Nutrient elements (Mg, P, S, K, and Ca) are indicated as means across all plant structures measured and across all fungal structures measured (±SE). Different letters indicate significant differences at P<0.05 between fungal cells of different of mycorrhizal species or plant cells associated with these species. Significant differences between fungal and plant structures are indicated by bold letters for P_(fungus/plant)._

In root cells associated with different mycorrhizal fungi, we also detected significant differences in nutrient levels (Table 1). The Mg concentrations were higher in ash than in beech root cells, but did not differ in root cells associated with different EcM species (Table 1). Roots colonized with *Lactarius* exhibited the highest P concentrations, whereas those associated with *Glomus* contained intermediate and those with *Cenococcum* or *Clavulina* low P concentrations (Table 1). *Cenococcum*-associated root cells contained higher S levels than those associated with other EcM. The S level was similar to that of *Glomus*-colonized root cells (Table 1). *Lactarius*-associated root cells contained high K levels, although the enrichment of this element was not high in the fungal structures of this species. The highest K levels were present in ash root cells. *Clavulina*-associated root cells contained high Ca levels, similar to the levels present in *Glomus*-associated ash root cells (Table 1). Overall, these data show significant differences in root nutrient element concentrations at a cellular scale of root tips associated with different symbiotic fungi.

To test whether relationships existed between the relative abundance of a nutrient element in plant structures (cells, cell walls) and fungal structures (cell, cell walls), we applied GLM. The dependent factor was the concentration of the element in the plant, categorical factors were: fungal species and localization (in  =  Hartig net, arbuscules and intracellular hyphae; out  =  Hyphal mantel, intercellular hyphae), and quantitative factors were all other elements in plant and fungal structures (walls, cells). For all studied nutrient elements (P, S, Mg, K and Ca) highly significant models were obtained (Table 2). The analysis underlined that “fungal species” was a significant factor in the GLMs for all studied elements (Table 2). However, none of the GLMs contained fungal nutrient element, but all combinations of plant-localized nutrients as significant factors (Table 2).

**Table 2:** Overview on the results of General linear Models for relative nutrient element concentrations in plant cells and cell walls.

Parameters	Mg in plant	P in plant	S in plant	K in plant	Ca in plant
Model	0.0000	0.0000	0.0000	0.0000	0.0000
F ratio	1266	433	852	897	37
R^2^(adjusted)	99.8	99.4	99.7	99.7	93.6
Species	0.0000	0.0000	0.0000	0.0000	0.0000
In/out	ns	ns	ns	ns	ns
Mg in fungus	ns	ns	ns	ns	ns
Mg in plant	na	0.0004	ns	0.0001	ns
P in fungus	ns	ns	ns	ns	ns
P in plant	0.0004	na	0.0000	0.0000	0.0000
S in fungus	ns	ns	ns	ns	ns
S in plant	0.034	0.0004	na	0.0023	0.0000
K in fungus	ns	ns	ns	ns	ns
K in plant	0.0001	0.0000	0.0000	na	0.0000
Ca in fungus	ns	ns	ns	ns	ns
Ca in plant	ns	ns	0.0000	ns	na

Information on the model parameters is given in the text, na  =  not applicable, ns  =  not significant

**Figure 5:**
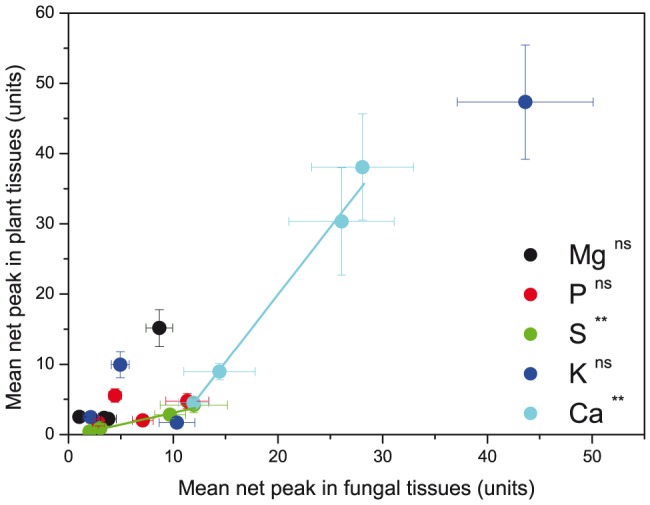
Relationship between subcellular element concentrations in plant and fungal tissues. ** indicate significant correlations with P<0.01. ns  =  not significant.

In previous studies, where correlations between ICP analysis of EcM and measurements of nutrient levels in the hyphal mantle by micro-analysis (EELS, EDX-SEM) were reported, subcellular compartments such as cell walls and intracellular nutrient concentrations were not distinguished [28]. Therefore, we also tested the relationship between the overall means of a nutrient element in the fungal structures with that in the plant structures (Fig. 5). For Mg, K and P no significant correlations were detected (Fig. 5). The plant S levels increased with increasing S levels in the fungi (Fig. 5). The plant Ca levels increased very strongly with those in the fungi, after exceeding a certain threshold in fungal tissues (Fig. 5).

## Discussion

### Arbuscular mycorrhizas are strong potassium accumulators

Forest trees can influence the chemical soil environment [2], as shown for ash and beech in the current study area [38]. In mixed stands with these tree species, the soil is less acidic than in pure beech forests because of the cation-pumping activity of ash [38], [39]. Ash roots contain higher cation levels than beech roots and ash leaf litter is nutrient-rich compared to that of beech [11], [12]. Since mycorrhizas are the main organ for nutrient uptake from soil and litter, a central question was whether AM fungi accumulate not only high P, as often demonstrated [23], [40], [41], but also high cation levels in comparison to EcM and whether these differences may influence the nutrient element levels of the associated root cells. A direct comparison is difficult because EcM and AM fungi differ fundamentally in the anatomical structure of their nutrient exchange interface [42]. Both mycorrhizal types increase their nutrient absorbing surface inside the root through branching of hyphae, either in the Hartig Net (EcM) or as intracellular hyphae inside the cells (AM). EcM store nutrients in the hyphal mantle [43], whereas AM form arbuscules, in which high P concentrations have been reported [23], [41]. Here, the EDX-STEM mappings demonstrated important differences in the nutrient distribution between fungal and plant structures. *Glomus* contained the highest K, Mg and P concentrations. The subcellular distribution pattern was particularly striking for K and Ca in the colonized ash roots because of their strong enrichment in the area surrounding the intracellular hyphae. The intracellular hyphae of *Glomus* are separated from the plant cytosol by the fungal cell wall and by the AM-typical periplasmatic membrane (interface compartment), an apoplastic compartment between the contact zone of plant and fungus [42]. This compartment consists of the membranes of both symbiotic partners, and contains pectin [44], which can bind Ca, K, and other cations and thus, could lead to the observed elemental signals. The K levels were also very high in the plant and fungal cells of *Glomus*-associated roots, whereas the Ca levels were low in those compartments. A striking result was that strong K enrichment, but not P as initially expected, was the major difference separating AM and EcM in a PCA. Therefore, our study suggests a significant role of AM in the cation balance of forest ecosystems with ash.

We confirmed that *Glomus* was enriched in P compared with EcM fungal structures; however, this difference was less clear for the associated plant cells. An early publication [45] demonstrated that approximately 90% of the accumulated P in EcM is located in the fungal sheath and not in the host tissues. We found approximately 2-fold higher P in fungal tissues compared to plant tissues. An important fungal P storage are polyphosphate granules, which have been identified in different mycorrhizal types, e.g., in the sheath and Hartig net of EcM in *Eucalyptus* and *Pinus*, in ectendomycorrhizas of *Arbutus* and in hyphae and vesicles of AM in *Liquidambar* [41]. In mycorrhizas, the granules are often also associated with K and Ca and supposed to regulate the exchange of elements between plant and fungus [46]. In our study the P-containing granules were detected the hyphal mantle of EcM as well as in the hyphae and arbuscules of *Glomus*. It is known that the arbuscules of AM have only a short lifetime of about 10 days [47] and release the stored elements into the plant cells, when they collapse. Thereby, the elements present in the granules may become available to the plant.

Although the fungal parts of the AM contained the highest P concentrations, the root cells of ash showed only an intermediate P concentration compared to those of EcM-associated beech root cells. Consequently, direct correlations between fungal and root cell concentrations of P were not observed. In general, the tissue concentrations of the elements are affected by the properties and binding capacity of the cell walls, by their mobility inside the tissues and by fungal or plant demand. Therefore, the finding that P, K, and Mg showed no correlations between fungal and plant structures may not be surprising. The observed relationship of S or Ca between fungal and plant tissues is notable and supports the findings of Rumberger et al. [28]. The reasons for divergent behavior and distribution patterns of the nutrient elements are unclear. Our data indicate that much of the variation is related to differences in the cell wall. Therefore, the wall properties, their modification by fungi and their potential role as reservoir for nutrient elements should receive more attention in future studies.

### Ectomycorrhizas exhibit profound interspecific differences for nutrient element acquisition

Our knowledge on functions of the major co-occurring EcM in forests is still limited. To address functional differences, we included three abundant colonizers of beech roots in the Hainich [13]: *Lactarius subdulcis*, a contact exploration type, with a hydrophilic hyphal mantle [48], *Clavulina cristata*, a medium distance exploration type [29], and the ascomycete *Cenococcum geophilum*, a short distance exploration type with a shiny black melanin-containing mantle [49]. Since mycorrhizal fungal species differ in their anatomy, mantle structures and mantle properties, and the distribution of hyphae in soil [50]–[52], it seems reasonable to assume that these species are functionally different regarding nutrient uptake, translocation and storage capacities. Our *in situ* analyses support this suggestion because we found significant differences among the elemental concentrations in the EcM taxa of our study.

*Lactarius* sp. and *Cenococcum geophilum* are frequent in many forests [25], [53], [54], while there are far less reports on *Clavulina cristata*. Rineau and Garbaye [30] found that *Clavulina cristata* became an abundant species in beech forests in response to liming. Interestingly, in the Hainich, this species occurs predominately in mixed beech forests with ash, i.e., under more alkaline conditions than in the acidic, pure beech forest [13]. The present study demonstrates the outstanding capacity of ectomycorrhizas with *Clavulina cristata* for Ca enrichment, not only in the fungal part but also in the root cells of the compound organ. This feature may have contributed to the higher Ca concentrations in beech than in ash roots [12]. Another EcM species able to accumulate high Ca concentrations in the mycelium is *Piloderma* sp. [55]. *Piloderma* sp. stores Ca in the cell walls of hyphae and the fungal mantle as calcium oxalate crystals [55]. It was suggested that, thereby, the hydrophobicity of the mantle was increased and that the Ca oxalate crystals served as protection against grazing microbes [55]. It is possible that the strong Ca enrichment in *Clavulina cristata* has similar functions. We observed that Ca increased in plant cells only after a certain threshold was exceeded in the adjacent fungal tissues. This finding suggests that Ca is initially predominately accumulated in fungal tissues. One may speculate that Ca is enriched in plant tissues after the uptake capacity of fungal tissues is exceeded. Whether and why the fungal cells have a higher retention capacity for Ca than plant walls needs further studies.

In a range of EcM morphotypes, whose nutrient elemental concentrations were analyzed by ICP-AES in beech-pine and spruce forests, *Lactarius* sp. assumed an intermediate position, while the nutrient concentrations were generally highest in *Xerocomus* and *Genea* and lowest in *Cenococcum* [25], [28]. *Xerocomus* and *Genea* are found on beech roots in the Hainich, but their abundance is very low [13]. The present data support that *Lactarius subdulcis* plays an important role for beech P nutrition because the root cells associated with this taxon accumulated the highest P levels among the EcM taxa studied, even slightly higher than those of *Glomus*-associated ash roots. This suggestion is also corroborated by the observation that *Lactarius subdulcis* is particularly active in oxalate secretion [26]. Oxalate, an organic acid, promotes mineral weathering and the release of phosphorus. It known that EcM hyphae contribute significantly to this process [56]. The finding that the P enrichment was specific for roots cells of mycorrhizas with *Lactarius subdulcis* and not for root cells associated with the other studied EcM species supports that fungal P translocation was responsible for P enrichment in the plant cells because the variation in the P concentration was related to fungal species on roots of beech trees grown in the same environment and thus to the same plant P demand.

*Cenococcum geophilum* exhibited the highest fungal S concentrations that were mainly located in the cell walls of the hyphal mantle. The chemical nature of S-containing compounds is unknown. It is possible that surface proteins such cystine-containing hydrophobins or binding of sulfate resulted in enhanced S concentrations in cell walls of this EcM species [58]. *Cenococcum* is also known for its ability to accumulate nitrogen in vacuolar bodies [24] and was found to maintain N uptake under drought stress [57]. Collectively, these results indicate functional differences of EcM with regard to plant nutrition, but also different benefits for the protection of root tips.

## Conclusions

Overall, this field study identified important functional traits that distinguish some abundant mycorrhizal taxa. The nutrient element levels suggested that *Cenococcum geophilum* plays a stronger role in plant S nutrition, *Lactarius subdulcis* in plant P nutrition, and *Clavulina cristata* in belowground Ca retention than the other analyzed fungi. Root cells of ash colonized by *Glomus* sp. contained outstanding K concentrations, but in contrast to our expectation not the highest P concentrations.

Ash trees are known to influence their soil environment by affecting the cation balance. Our results imply a major role of AM in this important ecosystem function. Currently, we cannot distinguish whether AM are responsible for high K acquisition from soil minerals or whether they have high capacities for K retention and efficiently recapture cations released from degrading leaf litter. This should be clarified in future studies. It should be emphasized that our results, obtained at a subcellular level, open new views on ecosystem functions and services at higher scales. Ash is a tree species with a wide ecological amplitude that gains importance as a component in mixed forests that are more suitable to withstand global climate change [59]. When forest tree composition is changed to cope with the challenges of global change, it is important to consider the consequences for symbiotic interactions and the edaphic environment.
